# Supplementary figures and images for: Integrated Physiological and Transcriptomic Analyses Reveal a Regulatory Network of Anthocyanin Metabolism Contributing to the Ornamental Value in a Novel Hybrid Cultivar of Camellia japonica
Source: Plants (Basel). 2020 Dec 7;9(12):1724. doi: 10.3390/plants9121724 (PMC7762260; doi:10.3390/plants9121724)

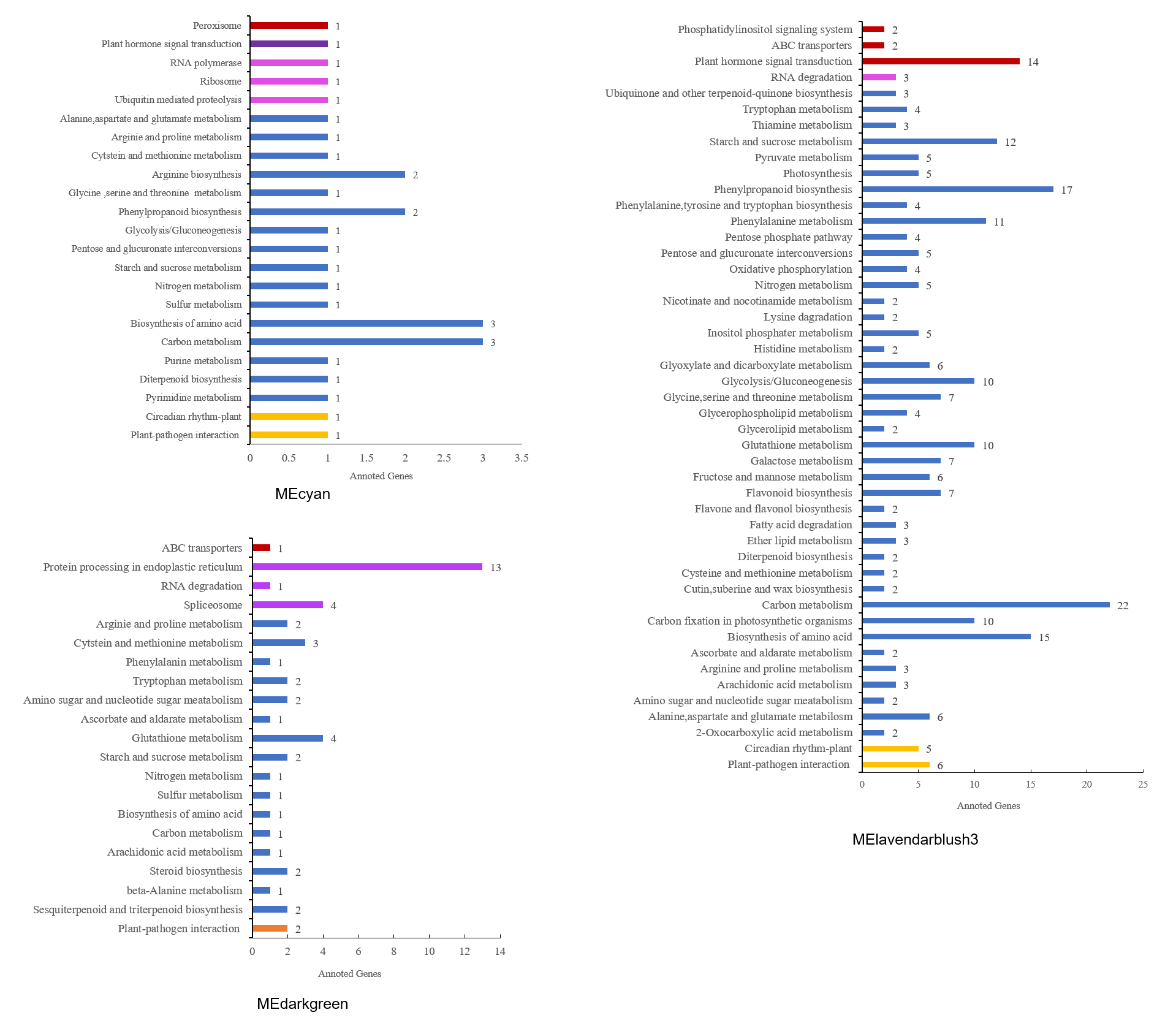

Supplement: Supplementary file 1 [file plants-09-01724-s001.zip › figS4.tif]

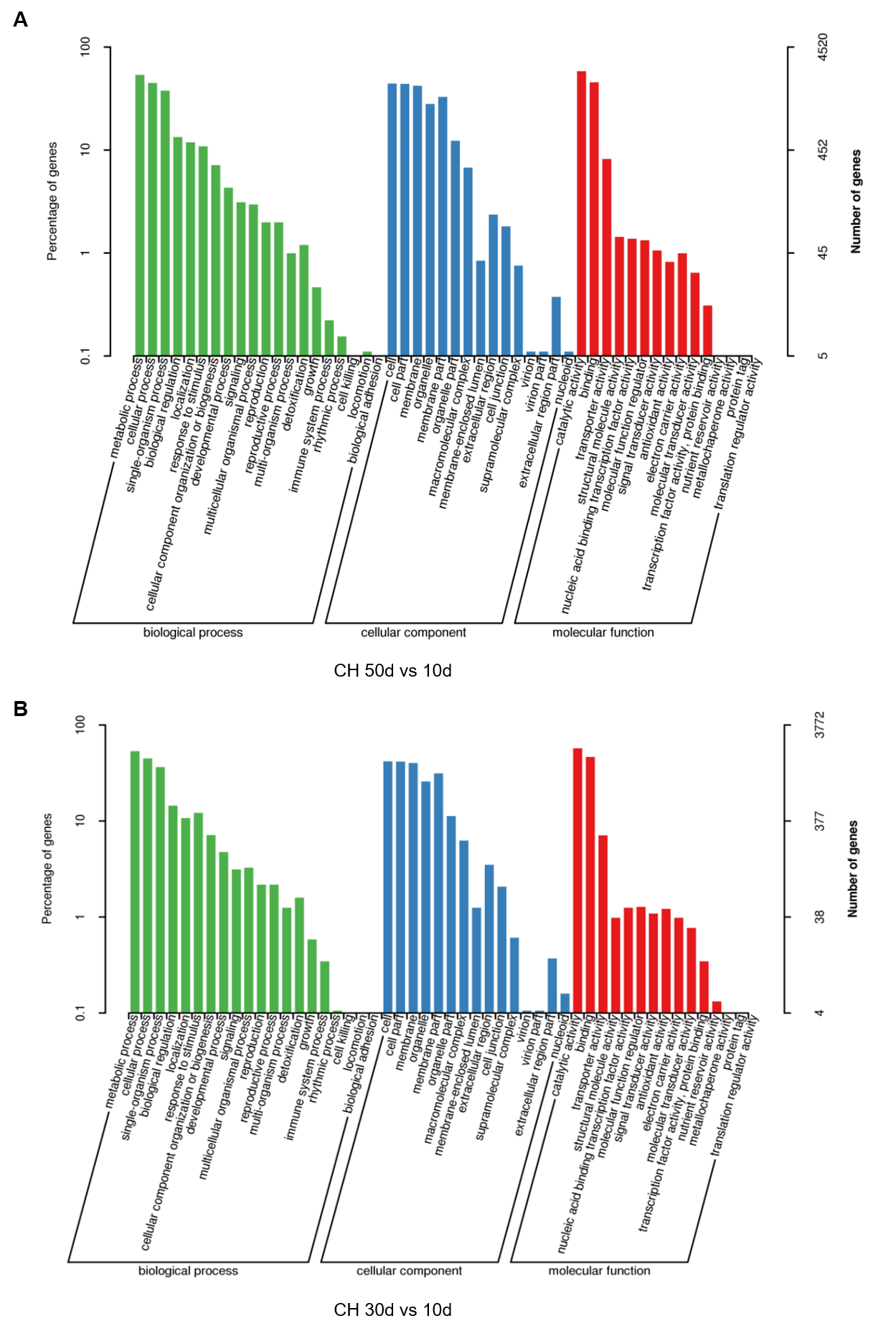

Supplement: Supplementary file 1 [file plants-09-01724-s001.zip › figS5.tif]

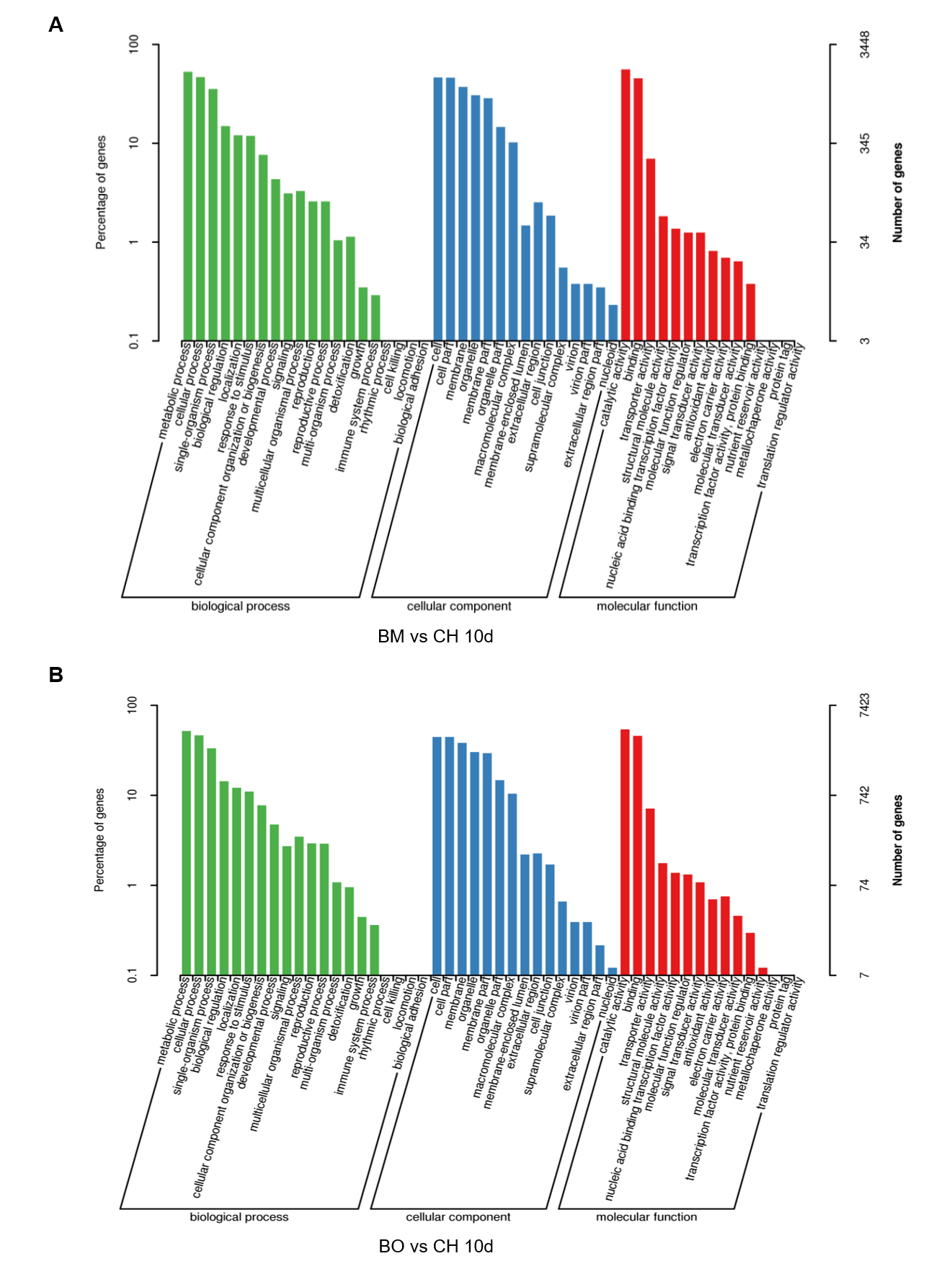

Supplement: Supplementary file 1 [file plants-09-01724-s001.zip › figS6.tif]

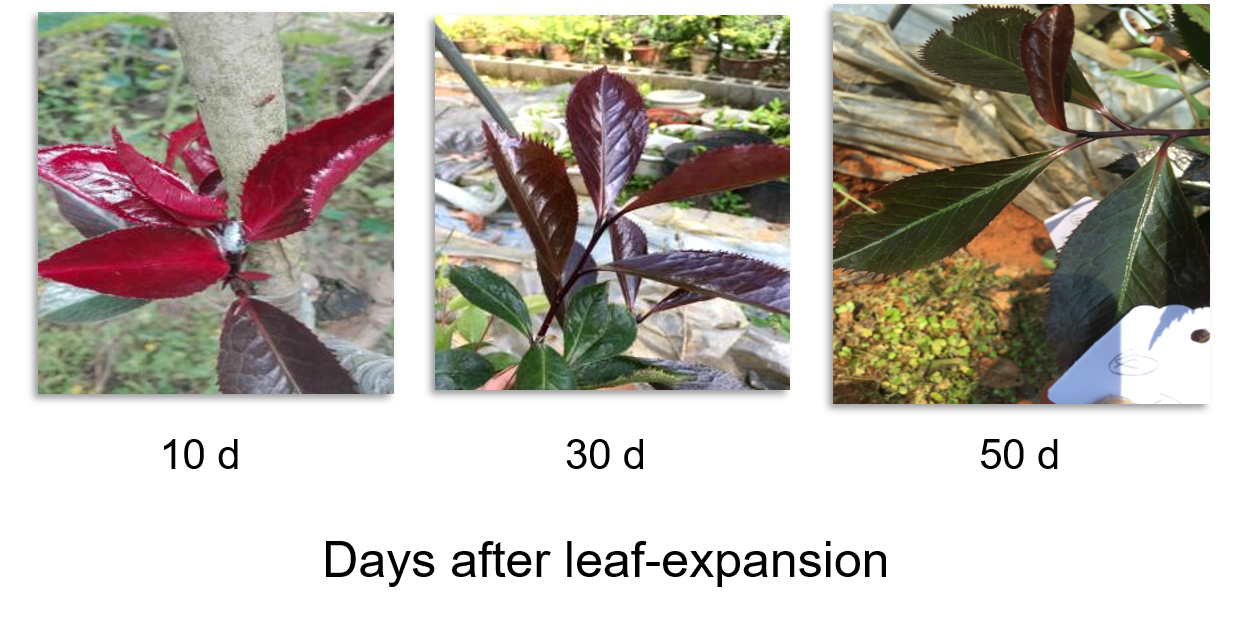

Supplement: Supplementary file 1 [file plants-09-01724-s001.zip › figS1.tif]

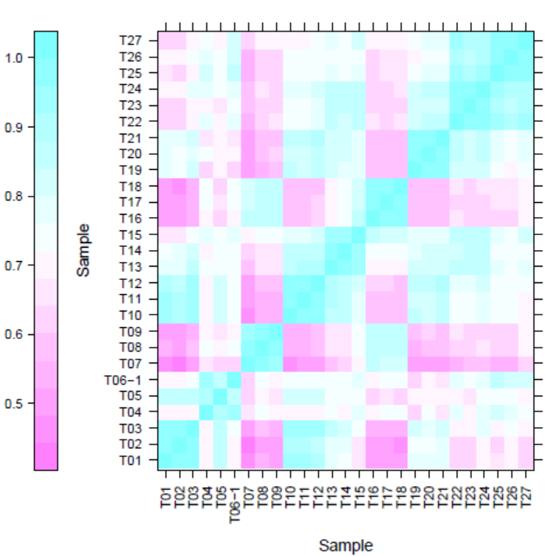

Supplement: Supplementary file 1 [file plants-09-01724-s001.zip › figS2.tif]

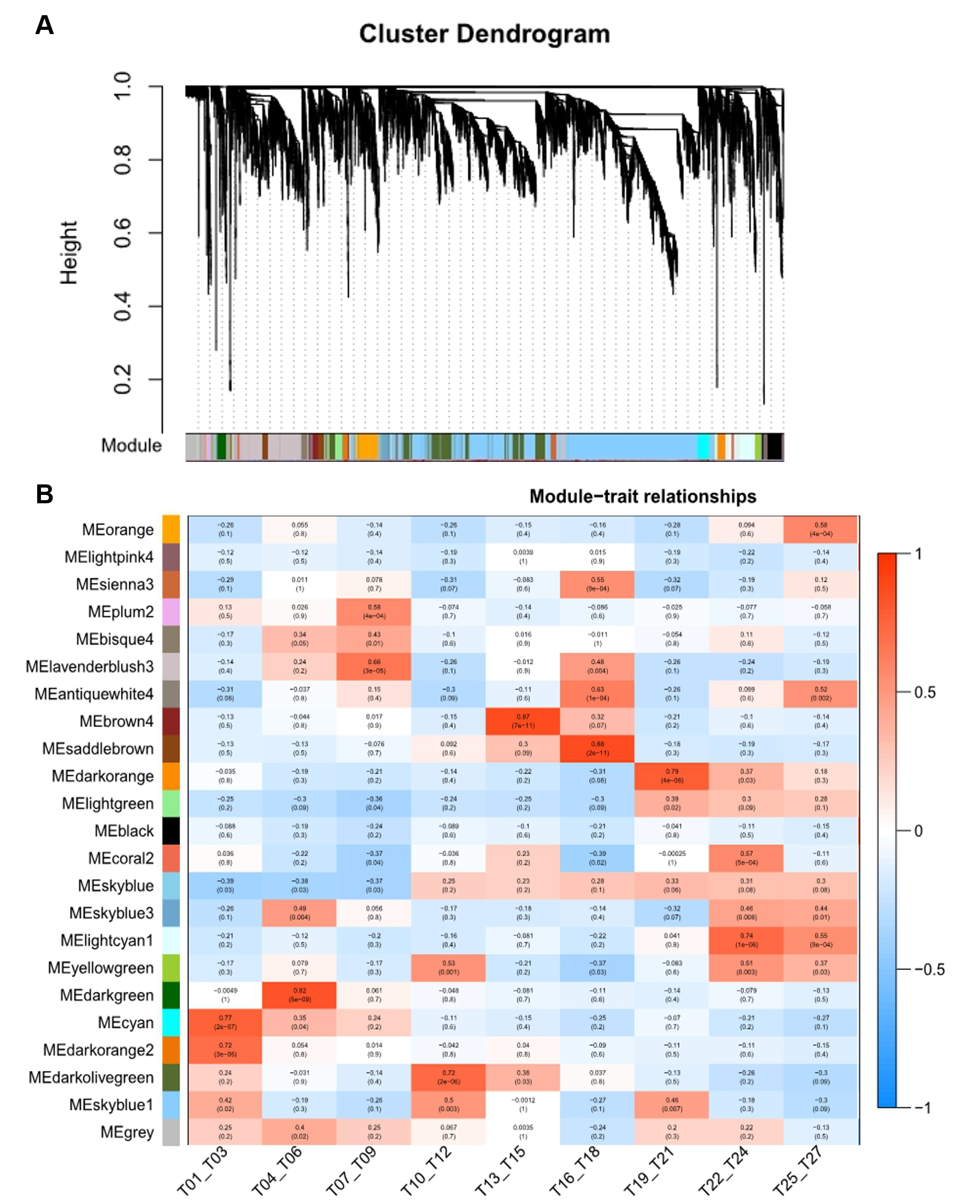

Supplement: Supplementary file 1 [file plants-09-01724-s001.zip › figS3.tif]
