# Supplementary material for: Integrated Physiological and Transcriptomic Analyses Reveal a Regulatory Network of Anthocyanin Metabolism Contributing to the Ornamental Value in a Novel Hybrid Cultivar of Camellia japonica
Source: Plants (Basel). 2020 Dec 7;9(12):1724. doi: 10.3390/plants9121724 (PMC7762260; doi:10.3390/plants9121724)
Supplement: Supplementary file 1 [file plants-09-01724-s001.zip › Supplementary Tables.doc]

**Table S1 Reads numbers of RNA-seq samples**

| Sample code | Sample name | Reads Number |
| --- | --- | --- |
| T01 | CH-10d-1 | 20,834,232 |
| T02 | CH-10d-2 | 21,579,501 |
| T03 | CH-10d-3 | 20,321,300 |
| T04 | CH-30d-1 | 20,542,865 |
| T05 | CH-30d-2 | 21,336,079 |
| T06-1 | CH-30d-3 | 22,939,997 |
| T07 | CH-50d-1 | 21,670,582 |
| T08 | CH-50d-2 | 21,002,658 |
| T09 | CH-50d-3 | 21,251,082 |
| T10 | BM-10d-1 | 22,379,047 |
| T11 | BM-10d-2 | 23,058,079 |
| T12 | BM-10d-3 | 22,297,337 |
| T13 | BM-30d-1 | 19,444,765 |
| T14 | BM-30d-2 | 21,323,173 |
| T15 | BM-30d-3 | 20,471,794 |
| T16 | BM-50d-1 | 20,985,150 |
| T17 | BM-50d-2 | 21,002,364 |
| T18 | BM-50d-3 | 22,065,689 |
| T19 | BO-10d-1 | 22,078,510 |
| T20 | BO-10d-2 | 22,386,414 |
| T21 | BO-10d-3 | 20,787,096 |
| T22 | BO-30d-1 | 20,542,849 |
| T23 | BO-30d-2 | 22,329,528 |
| T24 | BO-30d-3 | 20,600,140 |
| T25 | BO-50d-1 | 22,906,938 |
| T26 | BO-50d-2 | 21,332,466 |
| T27 | BO-50d-3 | 22,744,851 |

**Table S2 Number of functionally annotated unigenes**

| Anno_Database | Annotated_Number | 300<=length<1000 | length>=1000 |
| --- | --- | --- | --- |
| COG_Annotation | 16,902 | 3173 | 13,729 |
| GO_Annotation | 28,961 | 6366 | 22,595 |
| KEGG_Annotation | 16,943 | 3647 | 13,296 |
| KOG_Annotation | 24,250 | 4759 | 19,491 |
| Pfam_Annotation | 40,028 | 6174 | 33,854 |
| Swissprot_Annotation | 31,376 | 6199 | 25,177 |
| eggNOG_Annotation | 41,037 | 9662 | 31,375 |
| nr_Annotation | 42,112 | 10,128 | 31,984 |
| All_Annotated | 42,931 | 10,850 | 32,081 |

**Table S3 Numbers of differentially expressed unigenes (DEUs) in comparisons between growth stages**

| Cultivars | Comparison | All DEU | up-regulated | down-regulated |
| --- | --- | --- | --- | --- |
| CH | 50d vs 10d | 11130 | 6457 | 4673 |
|  | 50d vs 30d | 3700 | 1980 | 1720 |
|  | 30d vs 10d | 8692 | 4803 | 3889 |
| BM | 50d vs 10d | 13218 | 7548 | 5670 |
|  | 50d vs 30d | 4854 | 2836 | 2018 |
|  | 30d vs 10d | 6049 | 2957 | 3092 |
| BO | 50d vs 10d | 11638 | 6359 | 5279 |
|  | 50d vs 30d | 1404 | 751 | 653 |
|  | 30d vs 10d | 8375 | 4252 | 4123 |

**Table S4 Numbers of differentially expressed unigenes (DEUs) in comparisons between cultivars**

| Days after leaf-expansion | Comparison | All DEU | up-regulated | down-regulated |
| --- | --- | --- | --- | --- |
| 10d | BM vs CH | 8556 | 4785 | 3771 |
|  | BO vs CH | 12401 | 7113 | 5288 |
|  | BO vs_BM | 13698 | 7584 | 5670 |
| 30d | BM vs CH | 10483 | 5397 | 5086 |
|  | BO vs CH | 12092 | 6609 | 5483 |
|  | BO vs_BM | 14057 | 7712 | 6345 |
| 50d | BM vs CH | 7440 | 4106 | 3334 |
|  | BO vs CH | 13611 | 7402 | 6209 |
|  | BO vs_BM | 14031 | 7348 | 6683 |
